# Advancements in web-database applications for rabies surveillance

**DOI:** 10.1186/1476-072X-10-48

**Published:** 2011-08-02

**Authors:** Erin E Rees, Bruno Gendron, Frédérick Lelièvre, Nathalie Coté, Denise Bélanger

**Affiliations:** 1 Département de pathologie et microbiologie, Le Groupe de recherche en épidémiologie des zoonoses et santé publique, Université de Montréal, 3200 rue Sicotte, Saint-Hyacinthe, J2S 7C6, Canada; 2 Bruno Gendron, Consultant, 685 40ème avenue, Lachine, H8T 2G2, Canada; 3 Service de la biodiversité et des maladies de la faune, Ministère des Ressources naturelles et de la Faune du Québec, 880 chemin Sainte-Foy, Québec, G1S 4X4, Canada; 4 Direction de la santé animale et de l'inspection des viandes, Ministère de l'Agriculture, des Pêcheries et de l'Alimentation, 200 chemin Ste-Foy, Québec, G1R 4X6, Canada

## Abstract

**Background:**

Protection of public health from rabies is informed by the analysis of surveillance data from human and animal populations. In Canada, public health, agricultural and wildlife agencies at the provincial and federal level are responsible for rabies disease control, and this has led to multiple agency-specific data repositories. Aggregation of agency-specific data into one database application would enable more comprehensive data analyses and effective communication among participating agencies. In Québec, RageDB was developed to house surveillance data for the raccoon rabies variant, representing the next generation in web-based database applications that provide a key resource for the protection of public health.

**Results:**

RageDB incorporates data from, and grants access to, all agencies responsible for the surveillance of raccoon rabies in Québec. Technological advancements of RageDB to rabies surveillance databases include 1) automatic integration of multi-agency data and diagnostic results on a daily basis; 2) a web-based data editing interface that enables authorized users to add, edit and extract data; and 3) an interactive dashboard to help visualize data simply and efficiently, in table, chart, and cartographic formats. Furthermore, RageDB stores data from citizens who voluntarily report sightings of rabies suspect animals. We also discuss how sightings data can indicate public perception to the risk of racoon rabies and thus aid in directing the allocation of disease control resources for protecting public health.

**Conclusions:**

RageDB provides an example in the evolution of spatio-temporal database applications for the storage, analysis and communication of disease surveillance data. The database was fast and inexpensive to develop by using open-source technologies, simple and efficient design strategies, and shared web hosting. The database increases communication among agencies collaborating to protect human health from raccoon rabies. Furthermore, health agencies have real-time access to a wide assortment of data documenting new developments in the raccoon rabies epidemic and this enables a more timely and appropriate response.

## Background

**Figure 1:**
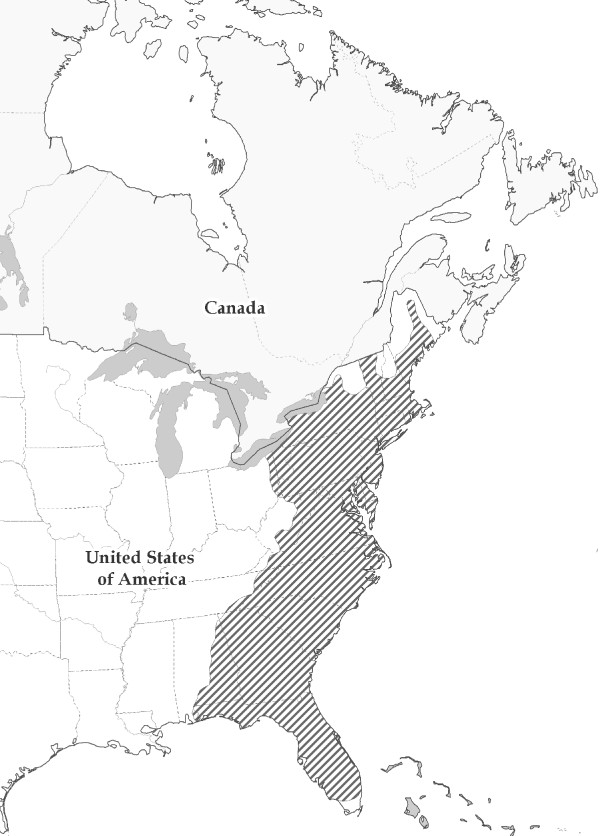
**Distribution of Raccoon Rabies**. Current distribution of the raccoon rabies variant in North America [15], as of June 2011 (Jesse Blanton, pers. comm.).

Rabies is a worldwide threat to public health, killing more than 55,000 people annually[1]. In North America, the raccoon variant of this virus has resulted in the largest wildlife zoonotic on record [2-4]. Though specifically adapted to raccoons, raccoon rabies can spillover into other mammals, including humans, through contact with infected saliva [5]. If not promptly treated, the rabies virus causes fatal encephalitis in nearly 100% of the human cases. Raccoon rabies was first observed in Florida in the 1940's [5] and then in the 1970's a second outbreak in West Virginia led to the current distribution of the disease in eastern North America [2,3] (Figure 1). Rabies is a significant public health concern because disease reservoirs occur in rural and urban areas of eastern North America, enabling rabid wildlife to infect humans directly or indirectly through domestic animals.

North American public health, agriculture and wildlife management agencies spend millions of dollars each year to protect the public and reduce raccoon rabies incidence [6,7]. The success of programs to control and prevent rabies depends on effective disease surveillance. In essence, disease surveillance requires sampling animals from populations of interest and diagnosing their disease status to estimate public health risk. More extensive surveillance may include collecting information on precise sample locations (e.g., georeferenced to a point location rather than a county centroid), sampling date, and biological characteristics of the animal sample (e.g., species, sex, general health condition). This information can then be used to generate more accurate and comprehensive epidemiological measures for quantifying the epidemic (e.g., prevalence, rate of spread) by accounting for factors that may affect the interpretation of surveillance data (e.g., biases in sampling methods). Ideally, surveillance programs enable a thorough understanding of the epidemic for estimating the health risk and for devising effective disease control strategies.

The effectiveness of surveillance programs to contribute data that are informative for risk assessment and disease control depends on the responsible agencies. Surveillance for diseases like rabies that affect public and animal health is often performed by multiple agencies. Even though the focus of an individual agency may be exclusively human, domestic animal or wild animal health, an understanding of all are needed for the protection of public health. A multi-agency approach for surveillance is advantageous by combining data from agencies with different mandates, typically resulting in a wider assortment of data collected over space and time. For instance, in Québec, five organisations participate to raccoon rabies surveillance. At the federal level is the Canadian Food Inspection Agency (CFIA), and the provincial agencies include the Ministère de l'Agriculture des Pêcheries et de l'Alimentation du Québec (MAPAQ), Ministère des Ressources naturelles et de la Faune (MRNF), Institut national de santé publique du Québec (INSPQ), and the Centre québécois sur la santé des animaux sauvages (CQSAS) at the Université de Montréal. The CFIA, MAPAQ, and INSPQ are domestic animal and human health organizations, while the MRNF and CQSAS are concerned with the management and health of wild populations. The CFIA diagnose animals collected during passive surveillance that may have exposed people or domestic animals to rabies, and additionally record information on sample timing and location. The INSPQ and regional public health agencies also participate in passive surveillance through collaboration with the CFIA. The MAPAQ is responsible for recording the location and frequency of citizens reporting rabies suspect animals, which may or may not result in an investigation to test disease status. The MRNF processes samples from their disease control programs (e.g., population reduction) and enhanced surveillance of samples collected to monitor the disease status of wild populations (e.g., road mortalities). The CQSAS conducts pathological analyses on samples and records information on weight, status of other diseases and parasite load, in addition to documenting typical biological attributes recorded for rabies surveillance (i.e., species, sex, age, rabies status^a^). Thus, there clearly can be a wide assortment of data available for designing effective disease management programs. However, to appropriately use data pooled from multiple agencies it is necessary to account for biases that can result from agency-specific sampling characteristics.

There are several ways in which agency surveillance data may differ. Firstly, agency-specific sampling designs may range in the spatial extent of their surveillance zone and the distribution and frequency of sampling events within the zone. Since rabies incidence varies across the landscape [8] and cycles in time [9,10], sufficient samples should be collected to capture the variation underlying the spatio-temporal characteristics of an epizootic. Failure to do so can bias the accuracy of epidemiological measures over space and time relative to the sampling design [11]. Secondly, the type of surveillance method used by agencies can lead to an under- or overestimation in observed prevalence. For example, surveillance through citizen notification surveillance detects a higher proportion of infected animals than found by trapping programs[12]. Citizens are more likely to report animals behaving abnormally from rabies infection than healthy animals, whereas traps typically capture healthy animals rather than those at the later stages of infection that are too sick to be attracted by or encounter traps. Thirdly, the geographic resolution of sample locations may vary among agencies, constraining the spatial scale for data interpretation. Sample locations may be geo-referenced by a global positioning device (GPS), municipal address, or to the centre of the associated town, county or census tract. It is always possible to georeference sample locations to larger spatial units, but not vice versa. Thus, the scale of the sample location affects the scale of the data interpretation. For example, rabies incidence data collected by the New York State Department of Health georeferenced to the street address are appropriate for accounting for the effect of human population density on observed rabies incidence per census tracts (e.g. [8]), but are too coarse for assessing the effects of micro-scale habitat features such as the interface of corn agriculture and forest on the rabies epidemic (e.g., [12]). Thus, the agency-specific differences that can affect data interpretation must be taken into account for the appropriate use of data.

Combining agency-specific surveillance data into one data repository is extremely beneficial for objective analysis and communication of surveillance findings. The design of the database can ensure that data are explicitly characterised by factors potentially affecting interpretation (e.g., level of geographic resolution for sample location). It is then the responsibility of the user to appropriately account for these factors. Also, use of a single data repository facilitates faster, coordinated access to data from agencies. This allows for more comprehensive analyses and communication of findings than possible with only an agency-specific database, and negates wasting human resources resulting from the creation of multiple independent and often redundant databases. The US pioneered an approach for housing rabies surveillance data into a single repository through the creation of RabID [13], an Internet accessible mapping application of rabies cases for public health agencies. Though advanced for mapping queried data by time period or species, there were no facilities for summarising queried data in other formats (e.g. plot, table) or for summarising a wider variety of surveillance data characteristics (e.g. type of sampling method, number of samples tested for rabies). Furthermore, the integration of data required a series of steps by personnel and automated processes to transfer, format and validate data. In 2008, the Raccoon Rabies Scientific Committee for the Government of Québec initiated development of their own next generation database, RageDB, to combine data from the CFIA, MAPAQ, MRNF, and CQSAS.

In this paper we outline the major advancements of RageDB: an automated data integration process from multiple agencies, next generation Web 2.0 applications for data-editing and mapping, and the storage of citizen participation data in rabies surveillance, to which, we comment on the value of including these data. We also discuss the role of RageDB in the creation of future spatio-temporal real-time web database applications.

## Results

### Multi-agency Foundation of RageDB

The Raccoon Rabies Scientific Committee is multi-sectorial and interdisciplinary. Committee members are from or have close association to all agencies participating in raccoon rabies surveillance in Québec. Therefore as facilitated through the committee, data storage and security needs for each agency stakeholder were taken into account during the development of RageDB.

### Database Components

**Figure 2:**
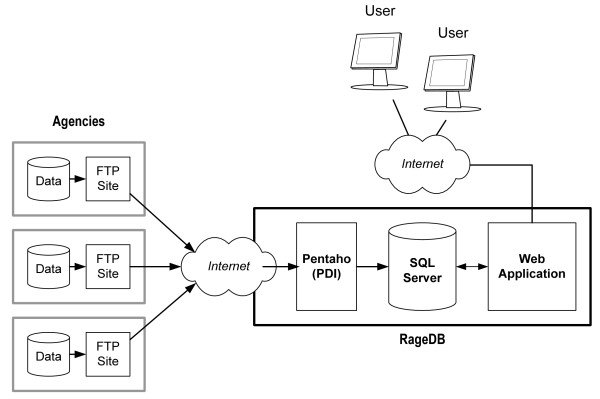
**RageDB architecture**. Overall architecture of data flow for RageDB, authorized users and participating disease management agencies.

RageDB consists of three main components (Figure 2). First, SQL Server (Microsoft Corporation, v2008, Redmond, WA, USA) relational database management system (RDBMS) is used as the storage tier. Second, agency-provided data are automatically integrated into SQL Server using the community (open-source) edition of Pentaho Data Integration platform (PDI; Pentaho Corporation, v4.1, Orlando, FL, USA). Finally, a Web application acts as the main user-interface for further data entry and editing, data exploration and extraction, as well as system administration (e.g., managing user accounts and privileges for data operations).

#### -Automated Data Integration

RageDB's automated data integration of multi-agency data is one of the key advancements in rabies surveillance repositories, as it demonstrates a simple but effective way of incorporating data from participating agencies with minimal investments from their information technology resources. The automated data integration process begins by having agencies extract relevant data from their own database systems and then depositing the data into their agency's secure FTP site. The agencies deposit their data at defined intervals and in an acceptable format of their choice (i.e., ASCII or CSV files, Microsoft Excel spreadsheets). PDI scripts, running under Microsoft Windows' scheduling facility, are then used to automatically access the data files from the FTP sites, transform the data to be compatible with RageDB's data structure, and then insert the results into the database. Success or failure notifications regarding the data integration are automatically sent to the RageDB administrators using PDI's logging and email functions. PDI scripts are also used to regularly backup the database to a remote location managed by the Groupe de recherche en épidémiologie des zoonoses et santé publique (GREZOSP), Université de Montréal in addition to the database's own daily backup.

#### -Data Validation

Data validation was critical for building RageDB to contend with the circumstance of combining data from multiple agencies that differed in their data management protocols. Since the overall architecture of the system allows multiple data-entry points through the web-interface and the Pentaho scripts, data validation could not be limited to the web-based data entry interface. Therefore, in addition to the normal data integrity constraints provided by the relational database management system, all data validation rules were implemented as small subroutines (called stored procedures) within the SQL Server. This advantageously eliminates the possibility of having an external process bypassing the validation rules.

Auditing and logging routines were also designed to track all modifications made to the database. These include the date and time of the modification and the values (i.e., past and current), and the identity of the user or automated process that made the modifications. Thus, by recording modifications in an audit table, RageDB administrators can restore data resulting from any erroneous edit and provide a history of all edits made to any piece of information.

#### -Summary & Cartographic Applications

The Web component of RageDB is composed of 2 main sections. The first is the data-entry and editing interface, a form-based website based on Microsoft's ASP.NET MVC framework (Microsoft Corporation, v.2.0, Redmond, WA, USA). The second section is the interactive dashboard, which is a pure HTML-based Web 2.0 application, developed using Javascript, and requiring no proprietary plugins. The dashboard was tested to be compatible with current Web browsers (e.g., Internet Explorer versions ≥7, Mozilla Firefox, Apple Safari, Google Chrome, Opera).

One of the key design goals in developing the dashboard was to provide users with a visually simple, yet attractive and interactive experience with the data, geared towards real-time data access for communication and decision-making. For more in-depth analyses or access to the complete dataset, the data-extraction facility of the main website can be used to output Excel or text files for subsequent data-processing in the user's software of choice.

**Figure 3:**
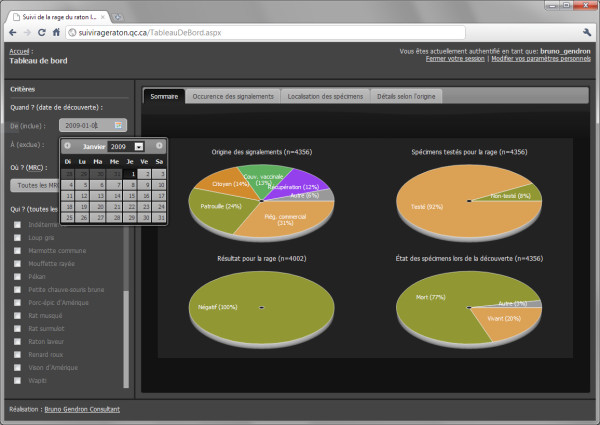
**RageDB dashboard**. Dashboard interface for querying and summarizing data by date, regional county municipality (MRC) and species, with pie chart format.

**Figure 4:**
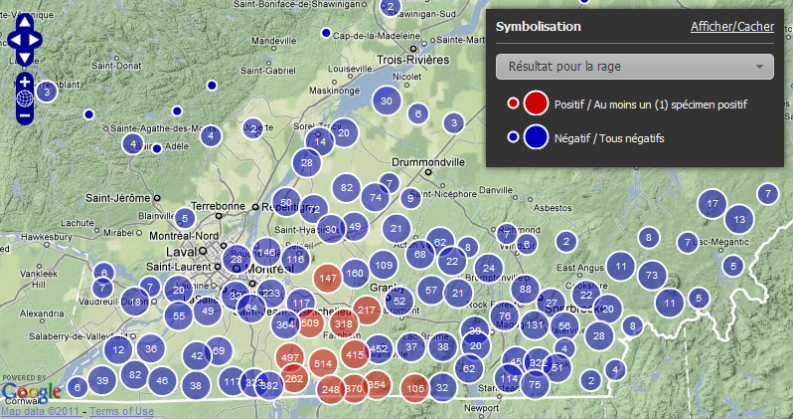
**RageDB dashboard cartographic output**. An example of cartographic output displaying the locations of animals tested for raccoon rabies in Québec as specified by species (raccoons) and time period (April 1^st ^2007 to April 1^st ^2011). Surveillance locations are depicted by red circles (contain ≥ 1 rabid sample) and blue circles (contain no rabid samples) and increase in size relative to the number of samples tested for rabies (as noted within the circle).

The dashboard screen is divided into 2 main areas (Figure 3). The sidebar on the left contains controls for filtering data by date, regional county municipality (MRC) and species. Users can select the summary format from the tabs on the main panel for presenting the surveillance data. The summary options are: (1) four pie charts showing the proportion of animals that were (i) collected per different sampling methods, (ii) tested for rabies, (iii) having a positive or negative rabies diagnosis, and (iv) discovered living or dead at the time of sampling; (2) a histogram for the frequency of citizens reporting suspect animals per week or month; (3) a table summarising the number of diagnoses and the rabies status of animals per sampling method; and (4) a map displaying sample locations (Figure 4). The map view uses OpenLayers' open-source Javascript mapping library with the Google Terrain layer as the background. Users can zoom in or out of the study area and choose to symbolize sample locations according to the disease status or health state of animals upon sample collection (i.e., alive or dead). To keep the map readable at any scale given the filtering options selected by the user, samples are clustered spatially and displayed as graduated symbols; symbol size increases relative to the number of samples clustered at the location. The clustering is handled dynamically on the server, according to the map scale and a static tolerance parameter that represents the minimum distance in pixels, at the given map scale, between each cluster.

To keep the user-interface responsive, the dashboard asynchronously communicates with the database through a Web Application Programming Interface (API). User interactions are sent to the server through the Web API and then forwarded to be processed by the SQL Server, with only the summarized results being returned to the Web application. Thus, bandwidth usage is minimized and the browser is prevented from processing large amounts of data.

#### -Benefits for Including Data from Citizen Notifications

Citizens in Québec are encouraged to report wild or domestic animals suspected of having rabies because this is the most effective surveillance method for detecting rabid animals [12]. Suspect animals include those that are found dead, behaving abnormally or with physical signs of aggressive encounters with other animals (e.g., lesions from bites). Citizens make a report through a phone number or website dedicated for rabies surveillance. The MAPAQ receives all citizen notifications and determines whether or not to dispatch an MRNF technician to collect the animal for rabies diagnosis. RageDB advantageously stores data on all citizen notifications, irrespective of whether the suspect animal was sampled for testing, though this distinction is also made in the database. Public health officials can use data on the total number of citizen notifications to measure public involvement in reporting suspect animals over time. This is important because citizens are more vigilante in participating at the onset of a known epidemic, but tend to become more complacent over time about disease presence. Therefore, regional monitoring of citizen participation can be used to identify areas where public participation is waning and then appropriate strategies can be devised to increase public participation (e.g., public service announcements in local newspapers and radio stations).

### Future Directions

RageDB is being continually developed to increase its value and functionality for public health. Participating agencies have already benefited from having quick access to all raccoon rabies surveillance data for aiding in policy development and research. Stringent security measures and user-restricted access makes RageDB a valuable tool for future storage of additional confidential data such as information on human and domestic animal exposures, and post-prophylaxis records. Furthermore, the success of RageDB for aiding management and research of raccoon rabies in Québec has initiated the MRNF, in partnership with the CQSAS, to develop a database for housing surveillance data from all wildlife diseases monitored in Québec. RageDB's data integration and storage architecture, and facilities for querying, summarising and mapping data are being used to guide the development of future wildlife disease databases.

## Conclusions

RageDB was built quickly (less than 3 months for the database itself and the web-editing interface) and inexpensively (approximately $30,000 Cdn) by using open-source technologies, simple and efficient design strategies, and shared web hosting. RageDB automatically integrates new surveillance data daily, provides a user-friendly interface for real-time viewing, summarising, and extraction of data. The collaborative effort from the multi-sectorial and interdisciplinary Raccoon Rabies Scientific Committee development team resulted in a multi-agency data repository that satisfied the needs of all stakeholders.

Having a common data repository for participating agencies is advantageous on many fronts. The value of surveillance data are increased because standardized validation and entry protocols ensure accurate data integration and storage, hence, maintains data accuracy for the duration of RageDB's existence. Easy access to view and analyse data from multiple agencies increases surveillance related communication among agencies and enables decision-makers to react rapidly to new developments. Increased communication among the agencies also facilitates multi-agency and inter-disciplinary collaboration for the protection of public health and the prevention of future rabies outbreaks. Furthermore, easy access to data collected from all participating agencies has enabled analyses that could not be accomplished by solely relying on agency-specific data. These include studies that estimated the risk of detecting raccoon rabies on the landscape [12], providing an evaluation of the efficacy of surveillance methods to detect rabies [12], and determining landscape factors associated with raccoon abundance (Houle et al., unpublished observations) and seropositivity of oral vaccination (Mainguy et al., unpublished observations).

The future of rabies surveillance and indeed, disease monitoring in general, will become increasingly more effective for informing disease control programs as web-based data repository technologies evolve. In time, we expect more agencies will realise the benefits of securely and accurately storing their data among multiple agencies for the benefits of sharing data to achieve the common goal of protecting public health.

## Endnotes

^a ^Since 2011 the Centre québécois sur la santé des animaux diagnoses the rabies status of animal samples using a direct rapid immunohistochemical test[14], for which the diagnosis is later confirmed by the Canadian Food Inspection Agency.

## Competing interests

The authors declare that they have no competing interests.

## Authors' contributions

ER is the administrator for RageDB and prepared the draft of this manuscript. BG is the systems architect and developer of RageDB. All authors are part of the RageDB development team and helped prepare this manuscript. All authors read and approved the final manuscript.
